# Comparing caregiver burden in owners of healthy dogs with and without limb amputation: a cross-sectional analysis

**DOI:** 10.3389/fvets.2026.1754268

**Published:** 2026-02-11

**Authors:** Janine Pryjmak, Yury Zablotski, Monika A. Mille, Susanne K. Lauer

**Affiliations:** LMU Small Animal Clinic, Centre for Clinical Veterinary Medicine, Ludwig-Maximilians-Universität, Munich, Germany

**Keywords:** canine amputation, caregiver burden, dog owners, prosthesis use, psychosocial support, veterinary counseling

## Abstract

**Objectives:**

In human medicine, limb amputation affects caregiver psychosocial burden. This study assessed caregiver burden in owners of dogs following limb amputation.

**Materials and methods:**

From March to September 2024, owners of healthy dogs with limb loss (> 6 months) and owners of healthy dogs without limb amputation (control dogs) were recruited via veterinary clinics, physiotherapy practices, university hospitals and social media to complete an online questionnaire. The 115-item survey included general questions on daily life and validated instruments assessing caregiver burden and psychosocial functioning: Zarit Burden Interview (ZBI), Perceived Stress Scale, Center of Epidemiologic Studies Depression scale, Generalized Anxiety Disorder 7-Item Scale, Quality of Life Enjoyment and Satisfaction Questionnaire. Descriptive statistics and group comparisons (Chi-Squared, Mann–Whitney U test and Students *t*-test) were performed in R.

**Results:**

Seventy-six owners of dogs with limb amputation (hindlimb: n = 35; forelimb: n = 41) and 74 owners of control dogs completed the survey (90.7% women; 70.7% aged 19–49). ZBI and other scores did not differ significantly between groups. Caregiver demographics (age, gender, relationship status, profession, number of caregivers) and dog-related factors (age at amputation, reason, leg affected, size, weight, adoption status) showed no significant effect on ZBI-scores. Higher ZBI-scores were reported when dogs could no longer hike (*p* = 0.05), and lower scores when caregivers received positive social feedback (*p* = 0.02). Prosthesis use (*p* = 0.02), low prosthesis acceptance (*p* = 0.04) or use of assistive devices (*p* = 0.02) were associated with higher caregiver burden.

**Conclusion:**

Most caregivers of canine amputees do not report elevated caregiver burden. However, functional changes in the dog and social or assistive factors were associated with caregiver experience.

## 1 Introduction

The phenomenon of caregiver burden in the context of veterinary literature has gained increasing scholarly attention in recent years (1–3). In this context, caregiver burden refers to the “level of multifaceted strain experienced by individuals responsible for the care of a family member and/or loved one over time” (4). In human healthcare, caregiving responsibilities are established sources of psychological, emotional and physical stress, frequently resulting in adverse health outcomes and diminished quality of life (5, 6). Elevated caregiver burden has been linked to psychiatric morbidity (7) including depression (8), anxiety (9) and suicidal ideation (10).

Emerging evidence suggests that owners of chronically ill companion animals may experience comparable psychosocial stress, as caregiver burden has been reported among those caring for dogs and cats with chronic conditions involving cardiologic, dermatologic, internal, oncologic, orthopedic, and neurologic disorders (1). Elevated burden has been associated with ethically complex decisions, such as consideration of euthanasia (11), as well as negative psychosocial consequences for pet owners (1, 12) and increased emotional strain within veterinary healthcare teams including burden transfer and burnout (13, 14). Studies in owners of dogs with cancer, epilepsy, behavioral disorders, and age-related cognitive dysfunction further demonstrate that chronic disease management can substantially affect owner psychological well-being and decision-making processes, reinforcing the clinical relevance of caregiver burden across diverse chronic and degenerative conditions (15–17).

In human medicine, limb amputation represents a profound life-altering event, that results in permanent functional impairment and diminished quality of life of the affected individual (18). Caregivers of human amputees frequently experience significant emotional, psychological, physical and financial stress, along with disruptions in social relationships (19). In small animal practice, limb amputation is a relatively common surgical intervention performed for a range of indications, including neoplasia, severe trauma, peripheral nerve dysfunction, ischemic necrosis, chronic infection, refractory osteoarthritis, and congenital limb deformities (20). Although this procedure is frequently used as a salvage intervention in veterinary practice, the psychosocial impact of limb amputation on dog owners remains poorly understood. Owners caring for a canine amputee that lacks normal locomotor function may experience increased caregiver burden. This assumption is supported by previous research showing that owners of dogs with osteoarthritis-related pain and functional decline exhibit elevated burden, which has been associated with euthanasia decision-making (2). While the underlying causes differ, chronic pain and progressive impairment in osteoarthritis versus the permanent loss of a limb in amputation, both conditions may increase caregiving demands and emotional strain. Thus, psychosocial challenges related to adaptation, altered mobility, and concern for the animal’s well-being may also arise in the context of limb amputation. A more thorough understanding of caregiver burden in this context is essential for improving support strategies for both pet owners and veterinary professionals. The objective of this study was to assess caregiver burden and psychosocial functioning in owners of dogs that had undergone limb amputation. Using a cross-sectional online survey, we evaluated levels of caregiver burden and associated psychosocial outcomes, including perceived stress, symptoms of depression and anxiety and quality of life. We hypothesized that owners of healthy dogs with limb amputation would report significantly higher caregiver burden and poorer psychosocial functioning compared to owners of healthy dogs without limb amputation. Furthermore, we hypothesized that the factors higher body weight and the use of prostheses, especially when poorly accepted by the dog, would be associated with an increased caregiver burden.

## 2 Materials and methods

### 2.1 Ethics and consent

The study protocol was approved by the Ethics Committee of the Faculty of Veterinary Medicine of the Ludwig-Maximilians-Universität München (approval number: AZ 384–27-12-2023; approval date: 10 January 2024). Prior to participation, prospective respondents were provided with a brief overview of the study and asked to confirm agreement with the data privacy policy. Eligibility criteria were presented and participants were required to confirm that they met these criteria before proceeding with the survey.

### 2.2 Study design

A cross-sectional online survey was conducted, employing validated and previously published instruments to assess caregiver burden and psychosocial functioning.

### 2.3 Reporting guidelines

The manuscript was prepared in accordance with the Checklist for Reporting Results of Internet E-Surveys (CHERRIES) (21).

#### 2.3.1 Study population

The study targeted two populations: owners of healthy dogs without limb amputation (control group) and owners of healthy dogs that had undergone limb amputation (amputee group).

#### 2.3.2 Survey development and pretesting

The questionnaire was developed based on prior research on caregiver burden and was designed and administered using Evasys, a web-based software for automated survey evaluation. Prior to distribution, the survey underwent independent review for functionality and clarity by two stakeholder groups: pet owners without veterinary training and veterinary professionals, including practicing veterinarians and doctoral students of veterinary medicine.

#### 2.3.3 Recruitment and access to the questionnaire

The survey was distributed online and accessed via a link or QR code. Recruitment occurred primarily through social media platforms and groups directed at owners of healthy, disabled and amputee dogs, supplemented by flyers at the Small Animal Clinic of the Ludwig-Maximilians-University Munich and an announcement on the clinic’s website. No incentives were offered. The survey remained open from March to September 2024. It comprised 99 items for the amputee group and 88 items for the control group, each including several validated instruments (see Section 2.5) and a general section.

Demographic variables included owner-related characteristics such as age, gender, relationship status, number of household members involved and professional background (categorized as human medicine, veterinary medicine, or other professions). Dog-related characteristics included age, body weight, sex, size (miniature, small, medium or large), age at the time of amputation, reason for amputation, affected limb and timing of adoption relative to the amputation. Additional variables addressed the caregiver’s perception of the dog’s quality of life, social feedback received from the environment, use and acceptance of prosthetic devices, use of assistive aids and the need to discontinue specific pre-amputation activities (e.g., daily walks, jogging, hiking, agility training, mantrailing). Based on the owner-reported body weight, dogs were classified into size categories as miniature (0–5 kg), small (>5–15 kg), medium (>15–25 kg), and large (>25 kg). Prosthesis acceptance was assessed based on owner report and referred to the dog’s tolerance and willingness to use the device during daily activities. Social feedback referred to reactions from the owner’s social environment, including family members, friends, acquaintances, and strangers encountered in public settings. The complete survey instrument is provided in the Appendix.

### 2.4 Participants

Eligible participants were German-speaking dog owners who had lived with their dog as a companion animal for at least 6 months prior to survey participation. No minimum or maximum age limits were applied to dogs in either group.

For the limb amputation group, dogs were required to have undergone unilateral limb amputation at least 6 months prior to participation. Dogs were excluded if owners reported the presence of additional acute or chronic medical conditions at the time of participation. Information regarding ongoing pharmacological treatment or structured rehabilitation was not systematically collected.

For the control group, dogs were classified as healthy based on owner report and were eligible only if no illness or veterinary treatment had occurred within the preceding 6 months. Health status was not independently verified by physical examination or laboratory diagnostics.

Across both groups, owners were excluded if they reported current mental health therapy.

### 2.5 Measures

Measurement tools were selected based on their established use in the human caregiving literature and their demonstrated psychometric properties, including reliability and validity. Representative example items are provided below solely to illustrate the content and scope of each instrument.

#### 2.5.1 Zarit burden interview

Caregiver burden was assessed using an adapted version of the Zarit Burden Interview (ZBI) (22). The original 22-item scale evaluates the perceived burden on a 5-point Likert scale from ‘never’ to ‘nearly always’ with higher scores reflecting increased levels of burden. Total scores above 20 indicate significant burden. The ZBI has demonstrated strong psychometric properties, including internal consistency (*α* = 0.9–0.91) and construct validity, as evidenced by a correlation of r = 0.73 with other measures of caregiver burden, quality of life and depression (23). For this study, the 18-item version adapted for pet caregivers was employed (1), which demonstrated excellent internal consistency (Cronbach’s *α* = 0.9). A cut-off score of 18 indicated elevated caregiver strain (see Table 1). Items assessed caregiving-related strain, including perceptions of stress arising from balancing pet care with other personal or professional responsibilities.

**Table 1:** Classification of caregiver burden based on the adapted Zarit Burden Interview (ZBI) score.

ZBI (adapted) score	Description
< 18	Normal
19–23	High average burden
24–33	Mildly elevated burden
34–43	Moderately elevated burden
> 44	Severely elevated burden

#### 2.5.2 Perceived stress scale

The Perceived Stress Scale (PSS) is a 10-item scale assessing how unpredictable, uncontrollable, and overwhelming individuals perceive their lives (24). Responses are given on a 5-point scale from ‘never’ to ‘very often’, with higher scores reflecting higher perceived stress. The scale has demonstrated acceptable internal consistency, with Cronbach’s alpha ranging from *α* = 0.68 to α = 0.78 (25). Additionally, evidence suggests that the PSS captures reliable variance beyond the general perceived stress factor (25). Perceived stress was assessed using items reflecting perceived lack of control and unpredictability in daily life, for example difficulties controlling important aspects of one’s life.

#### 2.5.3 Center for epidemiologic studies depression scale

The Center for Epidemiologic Studies Depression scale (CES-D) is a 20-item self-report instrument designed to assess depressive symptoms over the last week, using a 4-point Likert scale ranging from “rarely or none of the time” to “all of the time” (26). In adult populations, CES-D scores ≥ 16 indicate an increased risk for clinically relevant depressive symptoms, with higher scores reflecting greater symptom severity. The scale demonstrates acceptable convergent validity with clinical diagnoses of depression (*r* = 0.45), good internal consistency (Cronbach’s *α* = 0.82), and moderate test–retest reliability (*r* = 0.52) (27). Depressive symptoms were assessed using items reflecting core affective and cognitive features of depression, for example feelings of depressed mood and difficulties with concentration.

#### 2.5.4 Generalized anxiety disorder 7-item scale

The Generalized Anxiety Disorder 7-Item Scale (GAD-7) is a 7-item screening tool for generalized anxiety, scored from “not at all” to “nearly every day”, with higher scores indicating generalized anxiety (28). A score ≥ 10 yields sensitivity and specificity > 0.8 for the clinical diagnosis of generalized anxiety disorder (28), with high internal consistency (*α* = 0.89) across populations (29). Anxiety-related symptoms were assessed using items reflecting core features of generalized anxiety disorder, including nervousness or feeling on edge, excessive and difficult-to-control worry, and trouble relaxing.

#### 2.5.5 Quality of life enjoyment and satisfaction questionnaire – short

The Quality of Life Enjoyment and Satisfaction Questionnaire - Short (QLES-QSF) is a 16-item self-report instrument designed to assess perceived enjoyment and satisfaction across key domains of daily functioning, including mood, physical health, work and interpersonal relationships (30). Higher scores reflect greater overall quality of life. The QLES-QSF has demonstrated strong psychometric properties, including high internal consistency (*α* = 0.90) and excellent test–retest reliability (α = 0.93) (31). Negative correlations between the QLES-QSF and indices of depression and global improvement (*r* = −0.30 to −0.54) further support the validity of this measure (30). Quality of life was assessed using items measuring subjective well-being, including mood satisfaction over the preceding week.

## 3 Data analysis

### 3.1 Power analysis

Previous studies investigating caregiver burden in companion animal owners have demonstrated that scores of the adapted ZBI correlate with measures of depression, stress, quality of life, treatment plan difficulty and non-billable owner contacts, with reported correlation coefficients ranging from *r* = 0.31 to *r* = 0.59 (1, 32). Based on the *a priori* power analysis, a minimum sample size of 64 participants per group was required to detect a medium effect size (*r* = 0.5) with a power of 0.80 and a significance level of *α* = 0.05.

### 3.2 Statistical analysis

Descriptive statistics were calculated for all study variables. Group comparisons between owners of dogs with limb amputation and owners of control dogs were conducted using chi-squared tests for categorical data and Mann–Whitney U test or Kruskal-Wallis tests for continuous or ordinal data, depending on the number of groups. Normality was assessed using Shapiro–Wilk and Kolmogorov–Smirnov tests, both of which indicated non-normal distributions.

The chi-square test was utilized for the purpose of evaluating intercorrelations between two or more variables (e.g., gender, relationship status, number of caretakers, dog size). In this study, the Mann–Whitney U-test, a non-parametric statistical method, was employed to analyze the data, with the objective of assessing the differences between two groups (e.g., dog’s age, dog’s weight, Zarit Burden Interview, reason for amputation, amputated limb). In instances where the number of groups surpassed two, the nonparametric Kruskal–Wallis test was employed (e.g., dog size, dog sex).

Correlations between caregiver burden (ZBI) and measures of psychosocial functioning (PSS, CES-D, GAD-7 and QLES-QSF) were performed using Spearman’s rank correlation coefficient to show the relationships between psychosocial variables and caregiver burden. Results with a *p*-value < 0.05 were considered statistically significant. Data analysis was performed using R 4.2.1 (2022-06-23).

## 4 Results

### 4.1 Participant demographics

Demographic characteristics of the study sample are summarized in Table 2.

**Table 2:** Demographic features of the sample and group distinctions.

Demographic characteristics	Control dogs (*n* = 74)	Amputated dogs (*n* = 76)	*p*-values
Owners			
Gender (% female)	86.5	94.7	0.49
Relationship status (%)			
Single	68.9	41.1	0.02
Divorced	6.8	11.0	0.41
Married	24.3	47.9	0.02
Profession (%)			
Veterinary practice	29.7	21.1	0.02
Human medicine	5.4	26.3	0.001
Other profession	48.6	50.0	0.82
Number of caretakers (%)			
1	27.0	21.1	0.5
2	52.7	68.4	0.17
3	9.5	5.3	0.37
4+	10.8	5.3	0.25
Dogs			
Size (%)			
Miniature	2.7	5.3	0.41
Small	21.6	36.8	0.07
Medium	37.8	39.5	0.79
Large	37.8	18.4	0.03
Age (years), Mdn [range]	5.0 [5.0–7.0]	7.0 [6.0–9.0]	0.001
Weight (kg), Mdn [range]	23.0 [20.0–28.0]	19.0 [15.0–21.0]	0.03
Sex (%)			
Female	31.1	19.7	0.19
Female neutered	18.9	35.5	0.04
Male	27.0	9.2	0.01
Male neutered	23.0	35.5	0.13

#### 4.1.1 Pet owners

A total of 150 dog owners completed the survey, including 76 owners of amputee dogs and 74 owners of control dogs. The overall sample consisted predominantly of women (90.7%), with most participants aged between 19 and 49 years. Gender distribution, household size, and number of caregivers did not differ significantly between groups (all *p* > 0.05). Owners in the amputation group were employed significantly less often in veterinary medicine (21.1% vs. 44.6%, *p* = 0.02) and more often in human healthcare (26.3% vs. 5.4%, *p* = 0.001) compared to the control group. In addition, owners of amputee dogs were significantly more likely to be married (*p* = 0.02) and less likely to be single (*p* = 0.02).

#### 4.1.2 Dogs

Amputee dogs were significantly older (*p* = 0.001) and had significantly lower body weight (*p* = 0.03) compared to control dogs. Participating dogs represented a broad range of breeds and sizes, reflecting the heterogeneity of the study population (see Table 3). A significantly smaller proportion of owners of large dogs participated in the amputation group compared to the control group (*p* = 0.03). In the amputation group, significantly more owners of spayed female dogs (*p* = 0.04) and significantly less owners of intact male dogs (*p* = 0.01) participated compared to the control group. No significant group differences were observed in number of household pets (*p* > 0.05). In the amputation group, 53.9% of the dogs had forelimb amputations and 46.1% had hindlimb amputations. The most frequent reasons for amputation were trauma (52.6%), followed by unknown cause (18.4%), neoplasia (9.2%), postoperative complications (9.2%), refractory infection (6.6%), and congenital malformation (3.9%). Ownership had been established prior to the amputation in 53.9% of the dogs and afterward in 46.1%. Among owners of amputee dogs, 17.1% reported the use of prosthetic devices, and 36.8% the use of assistive aids (e.g., stroller, bicycle trailer, cargo bike). Among owners of dogs with limb amputation, canine quality of life was perceived as very good by 51.3%, good by 42.1%, and mediocre by 6.6%.

**Table 3:** Breed distribution of participating dogs by size category and study group.

Size category (kg)	Breed	Amputee group (*n*)	Control group (*n*)
Miniature (0–5)	Yorkshire Terrier	1	0
	Chihuahua	1	0
	Toy Poodle	0	1
	Mixed breed	2	1
Small (>5–15)	Dachshund	0	3
	Shetland Sheepdog	0	2
	Mixed breed	23	3
	Other breeds*	5	8
Medium (>15–25)	Belgian Malinois	2	1
	Mixed breed	22	13
	Other breeds*	6	14
Large (>25)	German Shepherd	1	5
	Labrador	1	3
	Mixed breed	8	6
	Other breeds*	4	14

*Other breeds include purebred dogs represented by a single individual.

### 4.2 Zarit burden interview

**Figure 1:**
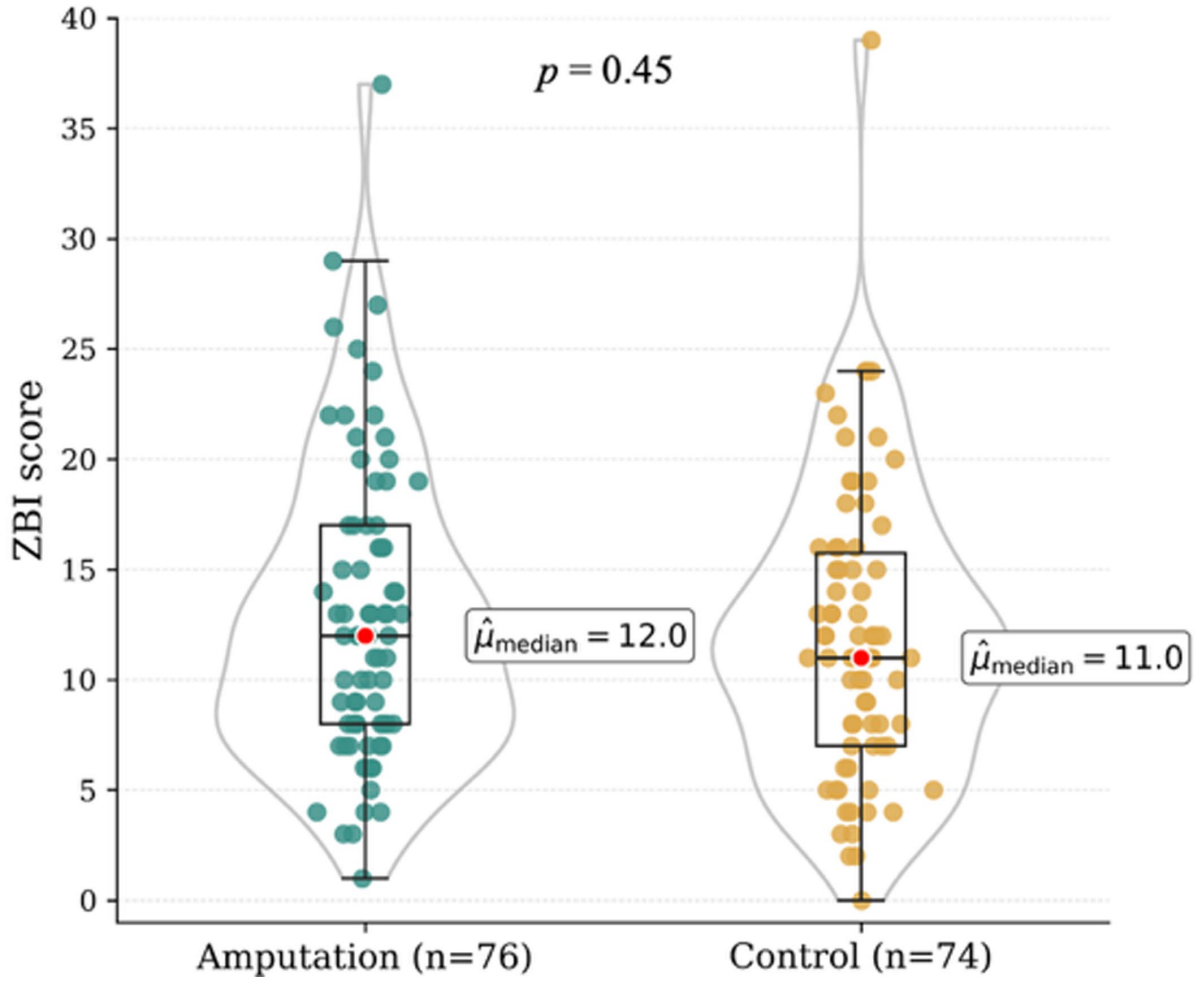
Median Zarit Burden Interview (ZBI) scores comparing caregiver burden between owners of amputee dogs and owners of control dogs.

**Figure 2:**
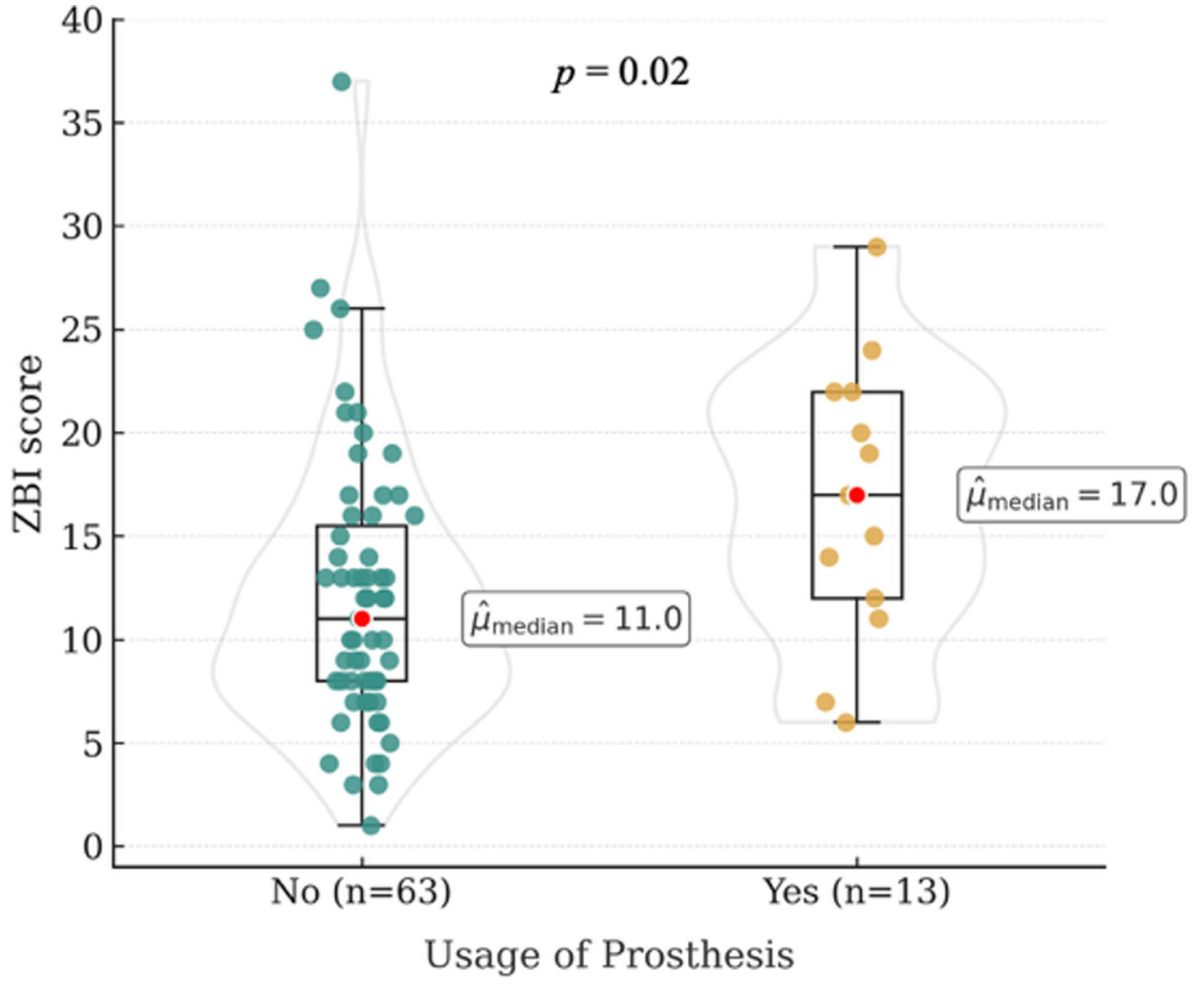
Median Zarit Burden Interview (ZBI) scores in relation to prosthesis use in dogs with limb amputation.

**Figure 3:**
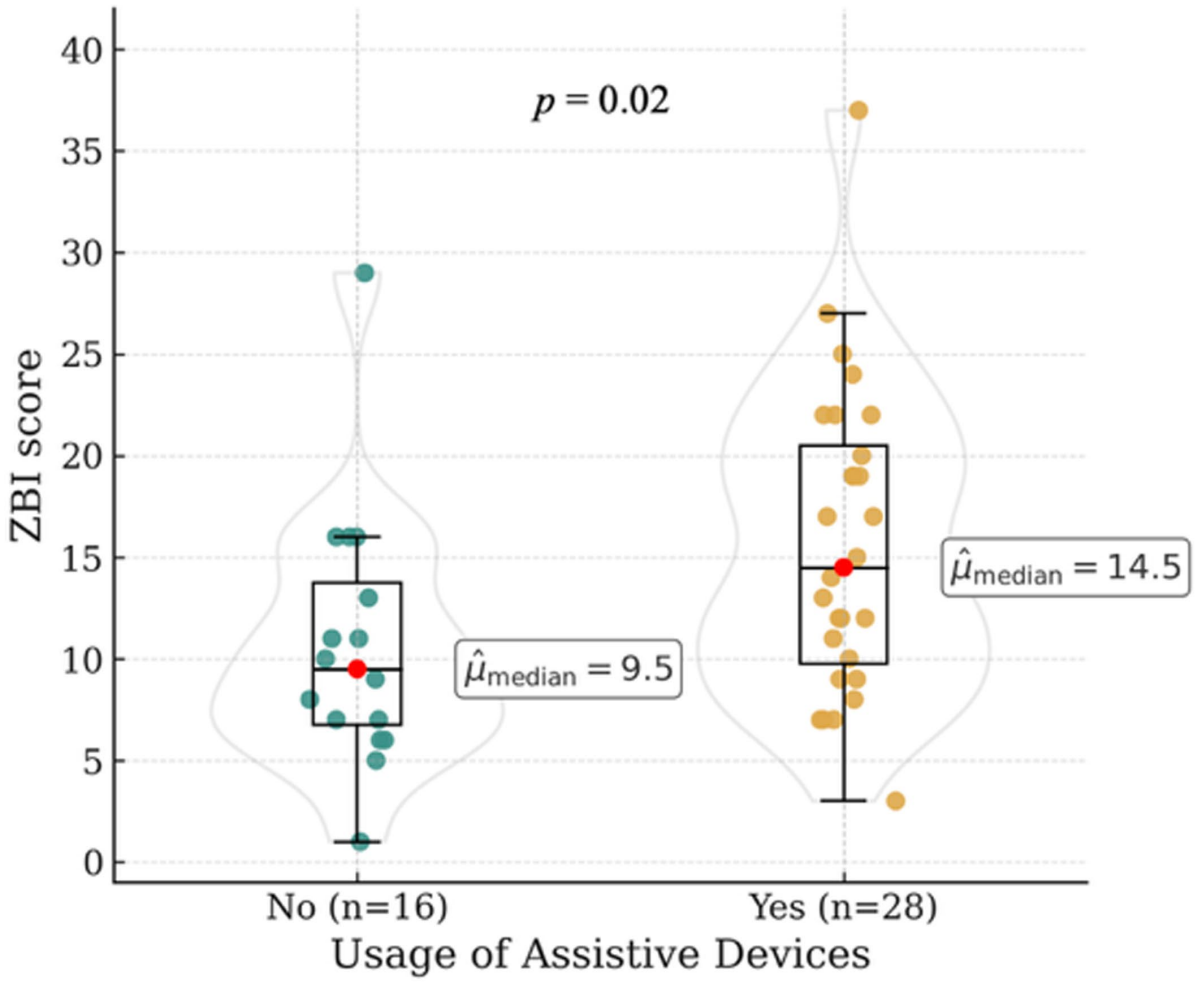
Median Zarit Burden Interview (ZBI) scores in owners of amputee dogs according to the use of assistive devices.

Median Zarit Burden Interview (ZBI) scores did not differ significantly between owners of amputee dogs (Mdn [range]: 12.0 [9.5–13.0]) and owners of control dogs (Mdn [range]: 11.0 [10.0–12.5]; *p* = 0.45) (see Figure 1). In the amputation group, ZBI scores were not significantly influenced by owner-related characteristics such as age, gender, relationship status, or professional background (all *p* > 0.05; see Table 4). Dog-related factors, including dog age (*p* = 0.51), age at the time of amputation (*p* = 0.08), reason for amputation (*p* = 0.08), limb affected (*p* = 0.51), and age at the time of amputation (*p* = 0.51) did not affect caregiver burden significantly. Owners of dogs that were no longer able to participate in hiking after amputation had higher Zarit Burden Interview scores (Mdn [range]: 17.0 [8.0–22.0]) compared to owners whose dogs were still able to hike thereafter (12.0 [9.0–13.0]; *p* = 0.05). Owners who reported positive social feedback from their social environment, including family members, friends and strangers, regarding living with an amputee dog had lower ZBI scores (Mdn [range]: 9.0 [8.0–12.0]) compared to owners who reported negative social reactions (Mdn [range]: 15.0 [12.0–17.0]) (*p* = 0.02). Caregiver burden was significantly higher among owners who applied prosthetic devices to their dogs (Mdn [range]: 17 [12.0–22.0]; *p* = 0.02; see Figure 2), reported poor prosthesis acceptance (9 of 17 owners; Mdn [range]: 19.0 [11.0–24.0]; *p* = 0.04), or used additional assistive devices to support mobility (Mdn [range]: 14.5 [11.5–19.0]; *p* = 0.02; see Figure 3).

**Table 4:** Distribution of Zarit burden interview (ZBI) scores according to demographic factors and group distinctions in owners of canine amputees.

Factor	ZBI Score (Mdn [CI_95_])
Owner age (years)	
19-29	11.0 [10.0–14.0]
30-39	10.0 [8.0–15.5]
40-49	13.0 [9.0–17.0]
50-59	12.0 [8.0–13.5]
> 60	10.0 [7.0–15.0]
Relationship status	
Single	12.0 [10.0–13.5]
Divorced	8.0 [5.0–13.0]
Married	12.0 [8.0–15.0]
Profession	
Veterinary practice	12.5 [9.0–20.0]
Human medicine	11.5 [8.0–16.0]
Other profession	11.0 [8.0–13.0]
Affected limb	
Forelimb	12.0 [10.0–15.0]
Hindlimb	12.0 [9.0–13.0]
Ownership timing relative to the amputation	
Prior	11.0 [8.0–14.0]
After	13.0 [10.0–14.0]
Reason for amputation	
Trauma	10.5 [8.0–13.0]
Unknown cause	13.0 [9.5–16.0]
Neoplasia	22.0 [8.0–24.0]
Postoperative complications	13.0 [10.0–17.0]
Refractory infection	8.0 [4.0–17.0]
Congenital malformation	13.0 [7.0–26.0]

### 4.3 Psychosocial outcomes

Perceived stress levels did not differ significantly between owners of control dogs and those of amputee dogs (*p* = 0.83). The Center for Epidemiologic Studies Depression Scale (*p* = 0.33), the Generalized Anxiety Disorder 7-item Scale (*p* = 0.64), and the Quality of Life Enjoyment and Satisfaction Questionnaire – Short Form (QLES-QSF; *p* = 0.11) likewise, did not differ significantly between groups. The mean scores obtained on the psychological tests administered to the two groups are presented in Table 5.

**Table 5:** Psychosocial outcomes in owners of control dogs and amputee dogs.

Psychosocial tests	Control dogs (*n* = 74)	Amputated dogs (*n* = 76)	*p*-values
Perceived stress scale (Mdn [CI_95_])	20.0 [19.0–21.0]	19.0 [18.0–20.0]	0.83
Center for Epidemiologic Studies Depression scale (Mdn [CI_95_])	5.0 [3.0–6.5]	4.0 [3.0–6.0]	0.33
Generalized Anxiety Disorder 7-Item Scale (Mdn [CI_95_])	3.0 [2.0–3.5]	2.0 [2.0–3.0]	0.64
Quality of Life Enjoyment and Satisfaction Questionnaire – Short (Mdn [CI_95_])	46.0 [44.5–47.5]	44.0 [42.0–46.0]	0.11

Caregiver burden correlated positively with perceived stress, depressive symptoms, and anxiety, as measured by the Perceived Stress Scale (PSS; *p* = 0.01), the Center for Epidemiologic Studies Depression Scale (CES-D; *p* = 0.001), and the Generalized Anxiety Disorder 7-item Scale (GAD-7; *p* < 0.001), respectively. In contrast, caregiver burden was negatively correlated with quality of life, as assessed by the Quality of Life Enjoyment and Satisfaction Questionnaire – Short Form (QLES-QSF; *p* < 0.001), indicating that higher caregiver burden was associated with lower satisfaction with daily life (Table 6).

**Table 6:** Spearman’s rank correlations between caregiver burden (Zarit Burden Interview) and psychosocial outcomes.

Psychosocial measure	Spearman’s *r*	*p*-value
Perceived stress scale (PSS)	0.29	*p* = 0.01
Center for Epidemiologic Studies Depression scale (CES-D)	0.37	*p* < 0.001
Generalized Anxiety Disorder 7-Item Scale (GAD-7)	0.44	*p* < 0.001
Quality of Life Enjoyment and Satisfaction Questionnaire – Short (QLES-Q-SF)	−0.47	*p* < 0.001

Positive correlation coefficients indicate higher caregiver burden associated with higher psychosocial distress. Negative coefficients indicate higher caregiver burden associated with lower quality of life.

## 5 Discussion

The aim of this study was to determine whether owners of dogs that had undergone limb amputation experience greater caregiver burden and reduced psychosocial functioning compared to owners of control dogs. Contrary to the initial hypothesis, no statistically significant differences were identified in caregiver burden, perceived stress, depressive symptoms, anxiety, or quality of life between the two groups. Nevertheless, caregiver burden correlated with psychosocial functioning across the entire sample. Within the amputation group, higher caregiver burden was reported by owners who used prosthetic or assistive devices for their dogs, particularly when prostheses were poorly accepted. In contrast, variables such as dog size and timing of ownership relative to the amputation did not significantly impact caregiver burden.

Previous veterinary research has predominantly focused on caregiver stress in chronic disease management, long-term treatment, or end-of-life decision-making, whereas the psychosocial implications of living with a canine amputee have remained largely unexplored. Several studies have examined owner-reported quality of life, satisfaction, and perceived outcomes in the context of orthotic devices, prostheses, or assistive mobility aids in dogs with heterogeneous orthopedic or neurologic conditions; however, these investigations were not specific to limb amputation and did not assess caregiver burden as a distinct psychosocial construct (33–36). Limb amputation presents a distinct set of challenges for owners, encompassing mobility adaptation, management of prosthetic devices, and social reactions to the dog’s visible disability. To the authors’ knowledge, this represents the first study to investigate caregiver burden and related psychosocial parameters in owners of canine amputees. A clearer understanding of these dimensions is essential to guide targeted caregiver support and to refine communication strategies within the practice of veterinary medicine.

Beyond individual caregiver burden, these findings highlight how dogs, as socially embedded companions, can shape owner experiences through social interaction when visible disability is present. Reactions from the social environment influenced caregiver burden, indicating that canine amputation affects not only daily caregiving routines but also social validation, stigma, and perceived support. This underscores that the impact of limb amputation extends beyond practical caregiving demands and includes changes in the owner’s social experience mediated by interactions with others.

In human healthcare, social support has been shown to mitigate emotional distress and promotes psychological adjustment among caregivers of individuals with chronic illness or physical disability (37–39). Consistent with this evidence, owners in the present study who received positive social feedback regarding their dog’s condition reported lower caregiver burden. This finding indicates that social reinforcement may buffer the emotional strain associated with caregiving after canine amputation. The results underscore the importance of social context in shaping owner experiences and indicate that supportive interpersonal environments may play a protective role in the adaptation process. In both human and veterinary contexts, the use of prosthetic and assistive devices introduces additional practical and psychosocial challenges (40, 41). In human medicine, dissatisfaction with prosthetic comfort, function, and maintenance has been associated with increased dependency, caregiver strain and reduced quality of life (42). Comparable difficulties have been described in veterinary patients, where prosthetic or assistive devices may cause skin irritation and restricted wear time which can result in limited adaptation (35, 43, 44). In the present study, owners who used prosthetic or assistive devices for their dogs reported higher caregiver burden, particularly when the animals showed poor acceptance of the device. This increased burden may reflect the added physical and emotional demands of prosthesis management, the time investment required, and unmet expectations regarding functional outcomes. However, as the clinical indications for prosthesis were not recorded, it remains unclear, whether the increased burden was primarily related to device-specific complications, the dog’s underlying functional impairment or the owner’s expectations. These factors likely interact and should be differentiated in future studies. In people, post-prosthetic rehabilitation programs have demonstrated benefits, yet comparable evidence in veterinary medicine remains scarce and warrants systematic investigation (45).

The findings of this study have practical relevance for veterinary professionals and adoption organizations supporting current or prospective owners of dogs with limb amputation. Although overall caregiver burden was not elevated, specific factors—such as prosthesis use, reduced physical activity, and limited social support—were associated with increased burden. These findings emphasize the importance of providing owners with realistic information regarding amputation outcomes, potential modifications to shared activities, and possible social reactions to amputation. Incorporating such psychosocial considerations into veterinary communication may help practitioners address client concerns more effectively and promote owner well-being. Greater awareness of the multidimensional influences on caregiver burden can contribute to more informed counseling and improved long-term caregiving outcomes.

Several limitations should be considered. Recruitment occurred primarily through social media platforms, which may have introduced selection bias and limited generalizability by disproportionately reaching individuals with medical backgrounds. Both human and veterinary healthcare professionals exhibit elevated rates of burnout, anxiety, and depressive symptoms due to occupational stressors, although the nature and contextual framing of these stressors differ between fields (46–53) Differences in professional background between groups, including a higher proportion of individuals working in human healthcare and a lower proportion working in veterinary medicine among owners of amputee dogs, may have influenced baseline perceptions of illness, disability, and caregiving demands, thereby potentially attenuating observable differences in caregiver burden between groups. In addition, owners with a background in human healthcare may be more likely to apply human-centered concepts of amputation and disability to canine patients, reflecting a degree of anthropomorphic interpretation. However, this possibility remains speculative and could not be examined in the present study.

Most participants were women aged 19 and 49 years, resulting in limited demographic diversity. This distribution mirrors trends in human caregiving research, where women are more frequently involved in caregiving roles and report higher emotional distress and burden than men (7). Consequently, the experiences of male dog owners may be underrepresented. Future studies should aim for a more balanced gender distribution and explore potential gender-related differences in caregiver burden and psychosocial outcomes. The cross-sectional study design precludes causal interferences or assessment of temporal changes in caregiver burden. Longitudinal studies are required to characterize how caregiver experiences evolve from decision-making through postoperative recovery and long-term adaptation. Comorbidities such as degenerative joint disease or behavioral disorders were not assessed and may have influenced caregiver burden. Likewise, individual differences in emotional attachment or coping style were not evaluated but may serve as moderating factors. External support received by participants, such as veterinary guidance, educational materials, or peer networks, was not recorded. Structured interventions, including owner education and psychosocial support resources, may mitigate caregiver burden and warrant further investigation. Although prosthesis use was associated with higher burden, the reasons for prosthesis prescription and the dog’s clinical condition were not documented. Prosthetic intervention may have been indicated in dogs with more pronounced functional impairment, which itself could increase caregiver demands. In addition, caregiver burden may have been influenced by factors not captured in this study, including variability in prosthetic device quality, anatomical eligibility for prosthetic success (e.g., residual limb length), whether structured rehabilitation was implemented, and the experience and qualifications of the individuals involved in device fabrication and fitting. Without detailed information on functional status, therapeutic goals, and prosthesis-related management, it remains unclear whether the observed burden primarily reflected prosthesis handling, underlying mobility limitations, or owner expectations. Finally, this study focused exclusively on dog owners and did not systematically assess or quantify the dogs’ impairments. Given species-specific differences in behavior and adaptation following limb amputation, future research should also examine caregiver burden among owners of other species, such as cats.

## 6 Conclusion

Caregiver burden did not differ significantly between owners of dogs with limb amputation and owners of dogs without limb amputation. However, higher burden was observed among owners whose dogs used prosthetic devices, particularly when the devices were poorly accepted and among those who reported a loss of shared activities such as hiking. In contrast, positive social feedback was associated with lower caregiver burden. These findings underscore the role of psychosocial and contextual factors in shaping caregiver experiences following canine amputation. Veterinary professionals should provide evidence-based guidance on prosthesis outcomes, counsel owners regarding potential adjustments in activity levels, and address social and emotional aspects of caregiving as part of comprehensive postoperative support. Incorporating these elements into clinical communication may help reduce caregiver burden and enhance the welfare of both dogs and their owners.

## Data Availability

Restrictions apply to the availability of these data due to institutional and ethical regulations. Data are available from the corresponding author upon reasonable request and subject to approval.
